# Do the Right Thing: A Privacy Policy Adherence Analysis of over Two Million Apps in Apple iOS App Store

**DOI:** 10.3390/s22228964

**Published:** 2022-11-19

**Authors:** Hamad Alamri, Carsten Maple, Saad Mohamad, Gregory Epiphaniou

**Affiliations:** 1 Warwick Manufacturing Group, The University of Warwick, Gibbet Hill Road, Coventry CV4 7AL, UK; 2 School of Cellular & Molecular Medicine, University of Bristol, Bristol BS8 1TD, UK

**Keywords:** privacy, privacy policy, app store, iOS privacy, Apple App Store

## Abstract

Mobile app developers are often obliged by regulatory frameworks to provide a privacy policy in natural comprehensible language to describe their apps’ privacy practices. However, prior research has revealed that: (1) not all app developers offer links to their privacy policies; and (2) even if they do offer such access, it is difficult to determine if it is a valid link to a (valid) policy. While many prior studies looked at this issue in Google Play Store, Apple App Store, and particularly the iOS store, is much less clear. In this paper, we conduct the first and the largest study to investigate the previous issues in the iOS app store ecosystem. First, we introduce an App Privacy Policy Extractor (APPE), a system that embraces and analyses the metadata of over two million apps to give insightful information about the distribution of the supposed privacy policies, and the content of the provided privacy policy links, store-wide. The result shows that only 58.5% of apps provide links to purported privacy policies, while 39.3% do not provide policy links at all. Our investigation of the provided links shows that only 38.4% of those links were directed to actual privacy policies, while 61.6% failed to lead to a privacy policy. Further, for research purposes we introduce the App Privacy Policy Corpus (APPC-451K); the largest app privacy policy corpus consisting of data relating to more than 451K verified privacy policies.

## 1. Introduction

It is widely recognised that almost all online services, including mobile apps, collect and share users’ personal information. Prior work shows that mobile apps access sensitive functionality and data (e.g., location, camera) that are not in their core functionality [1,2]. Research also indicates that users are generally unaware of the data collection and sharing practices carried out via their apps and often express surprise and concern when they learn about them [3,4]. This revelation results in app users being frustrated by app practices related to their privacy which, in turn, contributes significantly to the negative rating of apps [5,6].

In the absence of comprehensive global legislation, various organisations around the world have enacted privacy laws to enforce online services to provide privacy policies to outline the data practices conducted by their services. For instance, the European Union (EU) has developed and implemented the General Data Protection Regulation (GDPR) [7] which requires online services that are involved in the act of collecting and sharing personal and sensitive user data to post a privacy policy. Similarly, the US Federal Trade Commission in its Fair Information Practice Principles [8] urged online services to give notice of an entity’s information practices before collecting any personal information. Moreover, in its Mobile Privacy Disclosures report [9] it encouraged app developers to post a privacy policy. A similar requirement is contained in the California Online Privacy Protection Act [10] for online services accessible by California state residents. Hence, compliance with such regulations has become a requirement for every service running within the territorial scope of these laws.

Obviously, app stores are not an exception, and consequently global app stores such as Google Play and Apple App Store require app developers to provide a link to their privacy policies. As a general rule, both Google Play Store and Apple App Store instruct the developers to add privacy policy links to their apps store listing to comprehensively disclose how their apps collect, use, and share user data. The links of the privacy policies must be provided on the apps store listing page and within the app [11,12]. However, many studies reveal that more than half of the sample of their apps in different app stores fail to provide links to their privacy policies [13,14,15,16,17]. Furthermore, a considerable number of the policies demonstrated a lack of transparency and readability or contained misleading information [13,18].

It is not surprising that Apple has made heavy investments in branding itself as a privacy-centric firm. In fact, the company seeks to gain a competitive advantage by placing a large emphasis on privacy and protecting consumer information [19]. For instance, Apple App Store states that ’All apps must include a link to their privacy policy in the App Store Connect metadata field and within the app in an easily accessible manner’ [11]. However, we are not aware of the Apple App Store or any other app store distributor enforcing a systematic review on apps’ privacy compliance.

**Figure 1:**
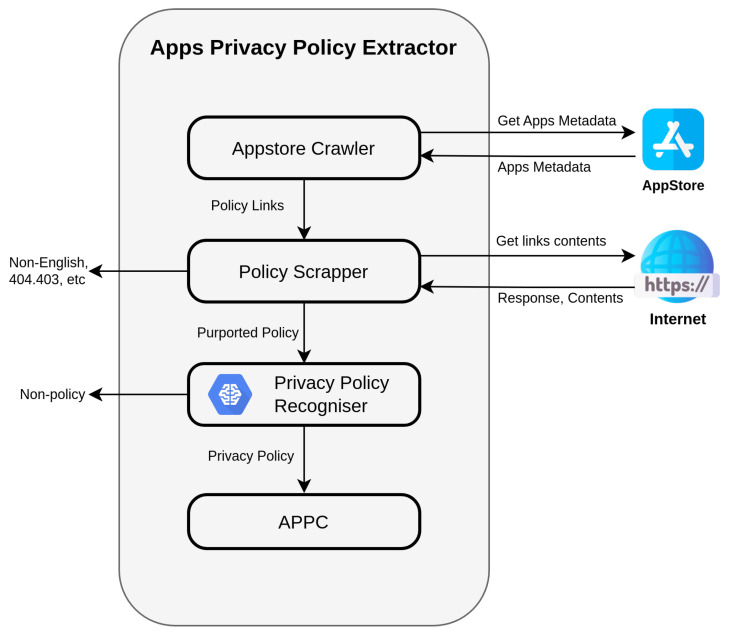
Overview of the App Privacy Policy Extractor.

In this study, we challenge the perception that Apple is a privacy-centric company by scrutinising the compliance of iOS apps in terms of providing privacy policies. For the purpose of launching this mission, we developed the App Privacy Policy Extractor (APPE), a system that evaluates the status of the apps’ privacy policy in the iOS app store. APPE not only determines the occurrence of the privacy policy link for every single app across the store, but it also examines and verifies the content of each provided link. APPE gives insightful information about the distribution and occurrence of the privacy policy across the entire iOS store and in every app category independently. Such a process may underline/identify the categories that need more attention from users when installing the apps, in addition to regulators and privacy organisations. The APPE system involves several phases including crawling the App Store, determining the availability of privacy policies links, scraping the alleged policy pages, and analysing their content. APPE is pictured in Figure 1 as an overview of its different components.

Throughout this paper, we seek to find answers to the following research questions:

1. What is the status of privacy policy distribution across the Apple iOS store?
2. How many apps provided attainable privacy policy links across the entire iOS store?
3. Is there a difference across app categories in providing attainable privacy policies?

The first and second questions are addressed in Section 4, where we display and discuss the results produced by APPE, while the third question is addressed in Section 5, where we make a detailed comparison between all app categories.

To the best of our knowledge, our work is the first and largest research initiative that has assessed the privacy status in the entire Apple iOS App Store, as well as providing a comprehensive overview of the privacy policies for each category of apps.

In this study, we make the following contributions:

- We introduce the App Privacy Policy Extractor APPE; a system that assesses the status of privacy policies across the entire iOS app store. APPE does not only reveal if apps provided links to their privacy policy (or not), it also follows the provided links, analyses their contents, and determines whether or not they lead to a valid privacy policy.
- An analysis of the entire iOS app store privacy source. Based on APPE analysis, we present an extensive privacy survey of over two million apps in the Apple iOS App Store. Our analysis finds that substantial numbers of the apps have privacy compliance issues. Specifically, almost 40% of the apps did not provide links to the privacy policy and over 60% of the provided links failed to lead to the privacy policy.
- We conduct a comprehensive comparative study between all app categories in terms of the content of the provided policy link, the error links, and the non-English links.
- We present the App Privacy Policy Corpus (APPC-451K), the most extensive App privacy policy corpus available in the literature. It consists of over 451K English privacy policy documents. APPC-451K will be available for the research community and can be used in further analyses or in machine learning experiments to produce useful information from, and relating to, privacy policies.

## 2. Related Work

This study is aware of several previous studies investigating the status of the privacy policies in the app stores ecosystem and constructing privacy policy corpus. Our work transcends the preceding work by investigating a vast number of apps in the iOS App Store and building a larger privacy policy corpus.

### 2.1. Assessing the State of the Privacy Policy in App Stores

The majority of the existing studies have examined a relatively small number of apps from quite a few different app categories. Authors in [13] assessed the availability, scope and transparency of privacy policies of mobile apps under Medical and Health&Fitness categories in the iOS App Store and Google Play. The finding of the study underlined the failure of developers to provide app privacy policies; documents which also lacked both transparency and readability when they actually were provided. In [14], the authors assessed the availability and the content of privacy policies of 369 mental health apps. The authors indicated that the majority of apps did not provide privacy policies, while many apps stated that users’ information might be shared with third parties. In a similar fashion, and with a focus on mental health apps [20] and depression apps [16], the researchers examined a different number of relatively small apps for privacy compliance. All studies reported missing privacy policies for a very high percentage of the apps and a lack of transparency around handling users’ data. Additionally, as part of a study examining the complexity of privacy policies for mental health and diabetes apps, the research reported that around 18% of the sample apps did not have links to their privacy policies and about 22.5% had broken privacy policy links [21]. Focusing on the privacy policies of Bipolar Disorder apps, this study [17] reported that not even a quarter of the collected sample of apps provided links to their privacy policies. In light of the recent COVID-19 pandemic and the emergence of contact-tracing apps, the authors in this study [22] have studied the privacy policies of the contact-tracing apps in both the Google Play Store and the Apple App Store. The study found that even though most of the apps are government-sponsored, 14.3% of 154 apps had no links to privacy policies, and the majority of the found policies were difficult to read. In a similar manner, the authors in [23] conducted a survey of the privacy policies of the Indian government-sponsored COVID-19 assistive apps. The study found that out of 63 public apps only 38% had app-specific privacy policies, while 11% had no privacy policies at all. It also found that in more than 51% of the apps the provided privacy policy did not lead to the privacy policies of those particular apps.

Focusing specifically on apps related to financial services, the authors in this study [24] performed an analysis of the privacy policies of mobile money services and compared them to policies from traditional US banking institutions. According to the study, 44% of these mobile money services had no privacy policy of any sort. Reviewing the readability and availability of the privacy policies of apps with the highest rankings that are specifically targeted at youth people, this research [25] reported that around 25% of 99 apps from Google Play store and Apple App Store are without links to privacy policy. In order to ascertain whether apps comply with the requirement of the Google Play store to have a privacy policy, the authors in [26] crawled Google Play store and collected 17,991 app metadata from all app categories. The study reported that approximately half of the apps (48%) lacked links to privacy policies.

Finally, and probably closer to our work, [27] conducted a study that analysed metadata involving one million apps from the Google Play Store. The finding states that only 50% of apps provided privacy policies links, and the created machine learning model highlighted some features that have directly and negatively affected the likelihood of apps having privacy policies (e.g., high rating, number of downloads, and in-app purchase). Unlike the previous studies, we conducted an intensive analysis for a more significant number of apps across every available app category in the iOS app store. Furthermore: (a) we reported the status of the links, (b) we examined the content and then (c) disclosed the content of all purported privacy policy links.

### 2.2. Constructing Privacy Policy Corpus

Typically, using Machine Learning (ML) techniques in text mining requires a relevant text corpus. In addition, the supervised learning demands a highly qualified annotated dataset to inform the model construction. Several studies reported using a privacy policy corpus when applying ML to privacy policies [28,29,30]. However, no information is given in those papers regarding the availability of the corpora or their construction. Likewise, the researchers in this work [31] reported using a corpus consisting of more than 130,000 privacy policies from websites and mobile apps to generate privacy-specific word embeddings that were used in privacy practices classification tasks. Nevertheless, neither the availability of the corpus nor its construction details are described in the paper. In order to automatically analyse the privacy policies of apps and detect violations with regard to Article 13 of the GDPR, the researchers in this work [32] have collected and annotated a corpus comprised of 304 privacy policies. These policies were further broken down into 36k sentences and labelled according to the label scheme developed in accordance with GDPR Article 13. Mapping between Permission and Privacy (MPP-270) [33] is another app policy corpus constructed from the privacy policies of 270 different Android apps. MPP-270 was labelled with dangerous permissions that allow apps to obtain sensitive information.

On the other side, The Usable Privacy Policy Project UPP [34] is an initiative started in 2013 that combines: (i) crowdsourcing, (ii) machine learning, and (iii) natural language processing (NLP) to build a practical framework based on existing natural-language privacy policies. The project has released OPP-115 [35] an annotated dataset of 115 websites’ privacy policies, wherein the annotation scheme was carefully designed, covered many privacy aspects, and was developed and applied by skilled domain experts. The opportunities for achieving the goal of analysing and processing privacy policies took a significant leap forward with the release of OPP-115 and enabled several applications to be developed [31,36]. ACL/COLING 2014 dataset [37] is an unannotated privacy policy corpus consists of 1010 privacy policies from the top websites ranked on Alexa.com, released by the project for research purposes prior to OPP-115. Moreover, the APP-350 corpus is also another dataset produced by UPP, which consists of 350 privacy policies of android apps which have been annotated by legal experts. Lastly, the UPP project [34] released the MAPS dataset that contains 441,626 links only for purported privacy policies [38]. APPC-451K, on the other hand, consists of more than 451K verified app privacy policy documents, making it the most comprehensive app privacy policy corpora in the literature as of this writing.

## 3. Approach

### 3.1. App Privacy Policy Extractor APPE

The App Privacy Policy Extractor APPE is a system that has been developed to facilitate our research. APPE was designed to serve three primary purposes: (1) assessing the incidence of privacy policies of the apps across the store; (2) analysing the content of every purported privacy policy link posted within apps’ pages; and (3) collecting the verified privacy policy document and constructing a comprehensive privacy policy corpus. The APPE pipeline involves three primary components: (a) an app store crawler, (b) a policy links scrapper, and (c) a privacy policy recogniser (PPR) system. An overview of the different components in our system is illustrated in Figure 1.

#### 3.1.1. App Store Crawler

The app store crawler is functioning by forming the categories and capturing apps metadata within each app category, and acquiring alleged privacy policy links of every app.

##### Forming the Categories and Capturing Apps Metadata

Typically, apps are structured into different categories within app stores (books, business, games, health, etc.) where developers can conveniently classify their apps. In this study, we have discovered twenty-four different categories in the iOS app store. Each category has two layers for classifying the apps; an alphabet layer consists of A-Z letters and a numeric layer for each alphabet. For example, the ‘Instagram’ app belongs to the ’Social Networking’ category and is categorised under the letter ’I’, but due to the large number of social networking apps having their names start with ‘I’, ‘Instagram’ categorised under numeric layer number 8, within the ‘I alphabet layer. The app store crawler starts by acquiring the categories of the apps first. Next, it grabs the first letter in the alphabet layer and the first number in the numeric layer under that specific letter. After that, it captures the app’s metadata, stores their information, and continues the process iteratively. By the end of this phase, information from over two million unique apps (2,010,403), listed into 24 different categories, had been obtained.

##### Acquiring the Privacy Policy Links

After composing the categories and obtaining all the apps’ information, the system then searches for the privacy policy link in the designated place within each app page. In order to identify the links of the privacy policies of the apps, we search the relevant URLs in the HTML element dedicated to privacy policies by the iOS App Store guidelines. If the HTML element contains a ‘href’ link labelled “privacy policy” it will be identified as a potential privacy policy URL. If the privacy policy link is provided, we classify the app in the (Apps provided Link to Privacy Policy) regardless of the content of the page, as it will be evaluated in a later phase. If the link is missing, the app will be classified as (Apps did not provide Link to Privacy Policy). Some apps return errors while crawling the store, which is probably due to its removal by the app developer or app store. Hence, any app that was not available at the time of crawling has been categorised as (App not available in-store).

#### 3.1.2. Policy Scrapper

This component is responsible for following the links to allegedly privacy policies, determining their validity (valid or broken link), and downloading the content associated with them. It utilises the Python Requests library to determine the validity of the links, and the real headless Google Chrome browser to scrape and download the contents of web pages. The advantage of using the real web browser over using the HTTP requests option is that it allows the content of the page including the JavaScript dynamically viewable content to be captured. We designed this component with the ability to handle the documents posted in HTML pages or posted in PDF, Microsoft Word (doc, docx), and txt format. We confirmed this with a number of randomly chosen policy’s links from all the 24 categories. We compared the Policy Scrapper results to content produced by the manual navigation of the same policy links. We found that the Policy Scrapper successfully scrapped all the content of the web pages, including the dynamically viewable elements.

The Policy Scrapper starts by checking if the link directs to a valid domain name first; if not, the link is categorised as a DNS Look Up error. Next, it checks the HTTP response status code. If the link returns a code other than 200 OK, the link is classified based on the error code it returned, such as: (a) 403 Forbidden (access denied), (b) 404 Not Found, and (c) 500 (Internal Server Error).

If the Policy Scrapper returned the HTTP 200 OK success status response code, which indicates that the request has succeeded, the system proceeds with parsing the entire content of the web page into plain text and checking the language of the page. If it is in English, it is sent to the Privacy Policy Recogniser PPR component to determine whether it is a privacy policy. If it is not in English, it is labelled as a non-English document. To determine the language of the documents we utilised an off-the-shelf Python package Langid [39]. Langid has been pre-trained over 97 different languages and has achieved high scores in language identification tasks. To verify the outcome of this component, we randomly selected items from each apps’ category and manually examined them. For example, for each app category we manually reviewed ten items from each error code such as 404 and 403. The reviewed items matched what was returned by the scrapper in all cases.

#### 3.1.3. Privacy Policy Recogniser PPR

To tackle the challenge of deciding whether each of the provided links leads to a valid privacy policy, we developed the Privacy Policy Recogniser PPR. The PPR is a core component in APEE, based on the Deep Learning DL approach. It is capable of broadly determining whether or not the submitted documents by app developers are English-language privacy policies on a large-scale. The two core components used to develop PPR model classifiers are LSTM and CNN, where LSTM captures the words correlation by treating the data as sequences, and CNN, in contrast, treats the data as chunks (sentences) sharing similar contextual correlation. We employed Glove [40] as a pre-trained model to extract the word embedding of our dataset in a method called transfer learning [41]. The classification layer of the model then follows this embedding. We used Python 3 as a programming language, PyTorch as the framework, and Torchtext for data processing. To find the optimal hyper-parameters for each algorithm, a randomised grid-search was run. For most of the classifiers the hyperparameter details were represented as follows: (a) Embedding size: 50-to-300, (b) Batch size: 16-to-512, (c) Learning rate: from 1e-2 to 1e-9, and hidden layers: 16-to-512. As part of our efforts to regularise the deep learning training process, and prevent the models from overfitting, we added dropout layers [42] to all deep learning methods we utilised.

##### Deep Learning Methods

In addition to CNN and LSTM, the core DL algorithms that we used to develop PPR, we also employed two variations of the core DL methods, namely, RCNN and LSTM-Attention.

The LSTM-based classifier used in our experiments consists of two LSTM layers followed by a dropout layer to alleviate overfitting. The last layer is a fully-connected layer that maps the output of the last hidden layer in the sequence to an output of size 2, which represents the policy and non-policy classes. In the LSTM-attention classifier, we incorporated an attention mechanism on top of the LSTM-classifier [43]. This additional layer is used to produce additional sources of information to guide the extraction of policy sentences into different vector representations.

When we developed the CNN-based classifier, we based it on the CNN sentence classifier proposed in [44]. The main concept is to perform convolution across privacy policy text to extract local contextual correlations. That is, each convolution filters out patterns from the document’s word embeddings representing keywords that are informative in recognising privacy policies. Our CNN architecture has three convolutional layers with multiple filters and filter sizes, followed by a max-pooling and fully-connected layer to assign the final labels (policy, non-policy). In our RCNN-based classifier, we developed a model architecture similar to the one proposed in [45]. We used a bi-directional LSTM to capture the left and right context of the current word vectors. In the following step, the current vector is concatenated with its surrounding vectors and passed through a non-linear function. After that, a max-pooling layer is applied, followed by a fully-connected output layer for classification.

In order to build a robust and accurate policy recogniser model, we developed several DL models and put them through extensive testing. We compared the performance of all models in all performance metrics, specifically: (a) accuracy, (b) recall, (c) precision, and (d) F-1 scores. The models with the best results were adopted.

As shown in Table 1, among the developed Deep Learning models, we adopted the LSTM model, as it achieved the best results in all performance metrics. Hence, in order to determine whether the documents are policies or not, we load the model and feed the pre-processed English documents produced by the Policy Scrapper component into the model. The documents are then classified into two classes: privacy policies and non-policy documents.

##### Data Set

Due to the supervised nature of our approach, a labelled dataset becomes a critical component of training and testing our deep learning techniques. As a result, we have manually created and labelled a dataset consisting of 9600 documents that had been divided into two categories: privacy policies and non-policy documents. The corpus used in developing the DL classifiers was manually created by the authors. We navigated through the downloaded English documents and searched and collected 200 privacy policies from each app’s category, resulting in 4800 privacy policy documents. In the same way, we manually collected 200 random non-policy documents from each category of apps. As a result, the final corpus contained 9600 policy and non-policy documents, which later were divided into a 80:20 ratio for training and testing purposes.

## 4. Results

### 4.1. What Is the Status of the Privacy Policy in the iOS App Store?

**Figure 2:**
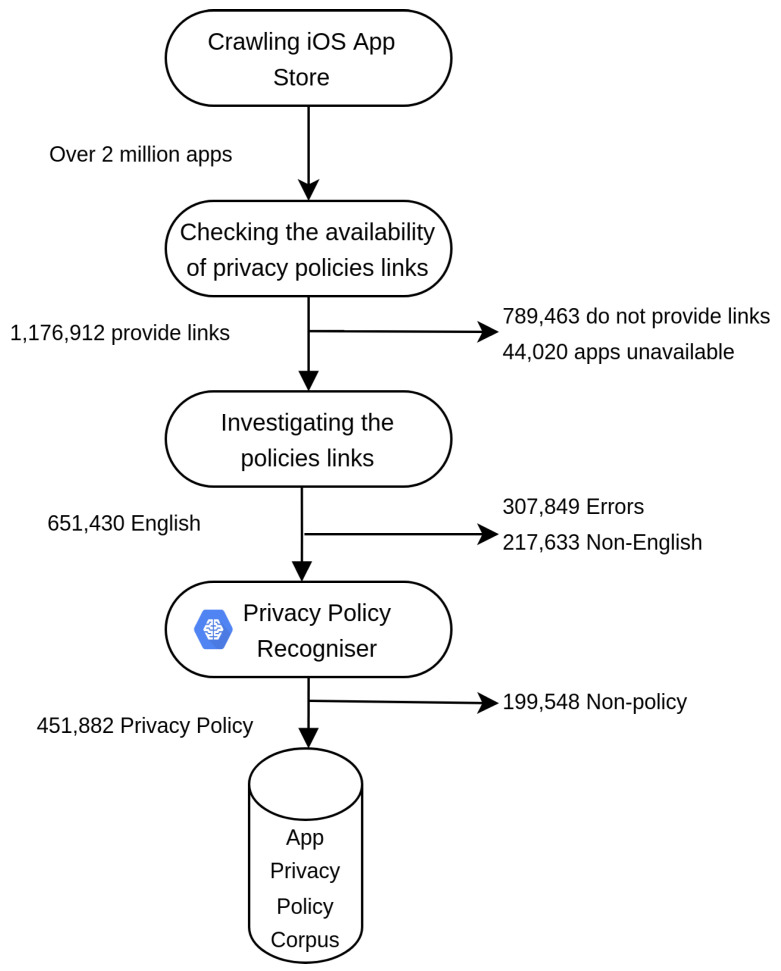
Processing pipeline of the App Privacy Policy Extractor.

In this section, we present the results of our store-wide evaluation of the distribution of the privacy policies and the investigation of the contents of the provided links. Figure 2 below illustrates the results of our work, as we progressed through the phases of APPE.

#### 4.1.1. Privacy Policy Distribution across Apple iOS App Store

**Figure 3:**
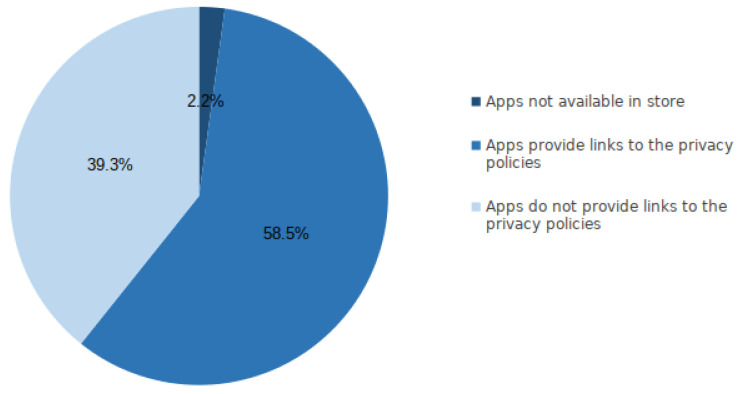
Overview of the availability of privacy policy links in iOS App store.

App developers are explicitly required to include privacy policy links on the designated app pages on Apple’s App Store platform [11]. Despite this obligation, a significant percentage of apps, as depicted in Figure 3, do not publish their privacy policies in the designated element within the store listing.

Based on our analysis, out of over two million crawled app metadata 1,176,912 apps, representing 58.5% of the crawled apps, provided links to their privacy policies. In contrast, 789,463 apps representing 39.3% did not provide links to their privacy policies. In addition, only 44,020 apps, which represent 2.2 percent of the total apps, were unavailable at the time of the crawl. We believe this omission may be due to removal of the apps by the App store or the app publishers. Though we evaluated the presence of privacy policies’ links on a much larger number of apps on the Apple iOS App Store, our result is broadly consistent with findings from a previous study, which showed that about 49% of apps did not provide links to privacy policies within the Google Play Store [38]. This result indicates that the Apple App Store does not differ from Google Play in terms of privacy policies provided by its apps. In general, this study on the Apple App Store, as well as the Zimmeck et al. [38] study on the Google Play Store, enables us to conclude that neither Google nor Apple are comprehensively enforcing their requirements for developers to disclose their apps’ privacy practices through providing privacy policies.

#### 4.1.2. Investigation of the Purported Policy Links

**Figure 4:**
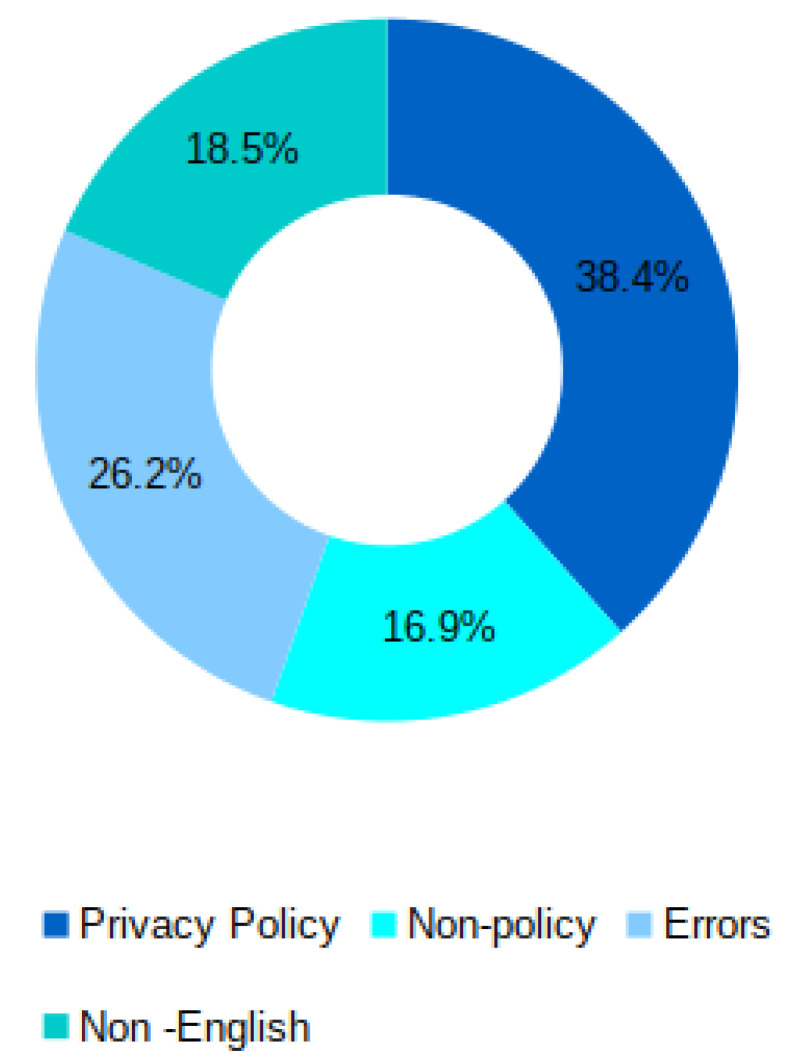
The results of examining every claimed policy link provided by the apps across the different categories.

Herein we present the results of investigating every purported privacy policy link. This process is done through two components of APPE, namely: (i) the Policy Scrapper and (ii) the Privacy Policy Recogniser. The details of the investigation into every app category is reported in Section 5. As demonstrated in Figure 4, our large-scale evaluation of the content of the provided links across the store, can be classified into four broad classes: (a) Privacy policy, (b) Non-policy, (c) Non-English, and (d) Errors. We will discuss each of these four classes in detail below.

##### Errors

Our results show that 26.2 percent of the provided links by apps in the different app categories returned connection errors when followed. In total, 56 different types of errors were encountered by our system. We found that the 404 Not Found error is the most common error, which was encountered more than 124K times, followed by the DNS Lookup error that has been exhibited by over 113K links. Both 404 and DNS Look UP errors constitute nearly 10% and 9% of the total provided links and nearly 40% and and 36% of the links that returned errors, respectively. Timeout and the 403 Forbidden errors were also encountered frequently, as over 35K links returned timeout and over 20K links returned the 403 error. These types of errors also represent a relatively big portion of both the total number and the error class of links. Additionally, our system encountered many server-side errors, such as 500 (Internal Server Error) 503 (service unavailable) and many different other errors. Table 2 shows the most frequent errors that occurred while examining the provided policy links. We intend to analyse the error types associated with every app category in our future work. Based on this result, it is evident that the App Store does not have any mechanism for verifying the legitimacy of the links that are supposedly provided by apps. It is obvious that the Apple App Store, at the very least, needs to implement automated tools to verify the validity of the privacy policy links, before they are provided by the apps developers.

##### Non-English

**Figure 5:**
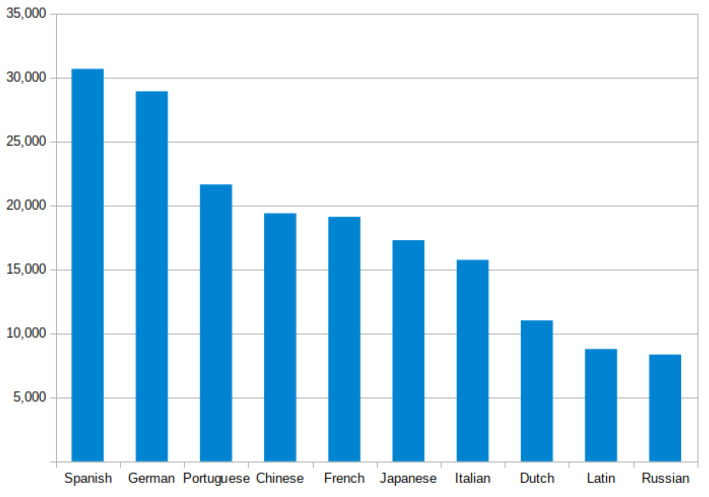
The distribution of links that refer to non-English language pages.

Since we are conducting our experiments in the UK, it is intuitively expected that the apps’ privacy policies will be written in English. Therefore, in this study we are only interested in the English privacy policies. After filtering out the non-English documents, we ended up with 651,430 English documents and 217,633 non-English documents representing 56.9% and 16.9% of the provided links respectively. As a matter of interest, our system identified 95 different languages, some of which appeared thousands of times while others appeared only a few times. As can be seen from Figure 5, there were more than 30,000 allegedly privacy policy links pointing to Spanish-language documents, making it the most prevalent non-English language in the App store. After Spanish, German comes in second, followed by Portuguese, as more than 20,000 links pointed to documents found in these languages. Moreover, the provided links led to thousands of web pages written in Chinese, French, Japanese, Italian and many other languages. Figure 5 illustrates the ten most common non-English languages detected by our system. As we conducted our experiments in the UK, the frequent presence of the policy links that lead to non-English policies elucidate the importance of enforcing the privacy-related regulations through the app developer communities around the world.

##### Non-Policy Documents

One of the other noticeable results produced by our system is the prevalence of links leading to non-privacy policy documents. The non-privacy policy provided links representing 16.9% of the total provided links. By analysing the content of the non-privacy documents, we found that most of the contents can be divided into three groups. First, the developer and the owner of the app homepages, where the provided links direct the users to the homepages of the organisation or the individual that owns the app. We should emphasise that we are not suggesting that these homepages lack links to privacy policies; rather, we believe that the provided link in the designated place for the privacy policy link should lead directly to the privacy policy, as required by the App store. This option is preferred to adding yet another burden on the user by requiring them to search for privacy policy links within the home pages. Additionally, it is most likely that the policies on the homepages are intended to cover the websites they are posted on. It should be noted that the Apple App Store has dedicated a specific location within the store listing for app developers to provide links to their homepages. Therefore, app developers posting their homepages into the element designated for the privacy statements may not even consider describing their app’s privacy practices. The second group is the irrelevant content, since numerous links are directed to web pages that contain short messages (e.g., site under construction) or inappreciable content. The last group is the empty content, where the links lead to web pages that contain no content to view.

##### App Privacy Policy Corpus

At the final stage, our APPE system has verified 451,882 privacy policy documents, a number which constitutes 38.4% of the total provided links. Hence, we call this large corpus of policy documents the App Privacy Policy Corpus (APPC-451K). It consists of over 451K policy documents from 24 different app categories. The privacy policies in our corpus have an average sentence count of 68 sentences per policy and an average of 26 words per sentence. They also have a mean policy length of about 1845 words per policy, and standard deviation of about 1668. The word count of the policies in this corpus ranges between 8k and 22k words. Yet, we intend to conduct a more comprehensive analysis on the content of the corpus in future work. The App Privacy Policy Corpus APPC-451K will be released for further research under the creative commons license.

## 5. Comparative Analysis of App Categories

In the previous section we outlined a broader analysis of the store-wide distribution of privacy policies. In this section, we aim to provide insightful information about the policies in every app category. We present our analysis of the results of comparing the different app categories in terms of: (a) availability of privacy policies links, (b) verified privacy policies, (c) non-policy contents, and (d) the encountered errors.

### 5.1. Availability of Privacy Policy Links

As mentioned earlier in the paper, about 58 percent of over 2 million apps provided links to a privacy policy. Yet, since our system discovered 24 different app categories in the store, it is interesting to explore the availability of privacy policy links in every app category. This initiative will highlight the categories that have less availability of policy link access, which might help users to pay more attention when downloading apps from these categories.

**Figure 6:**
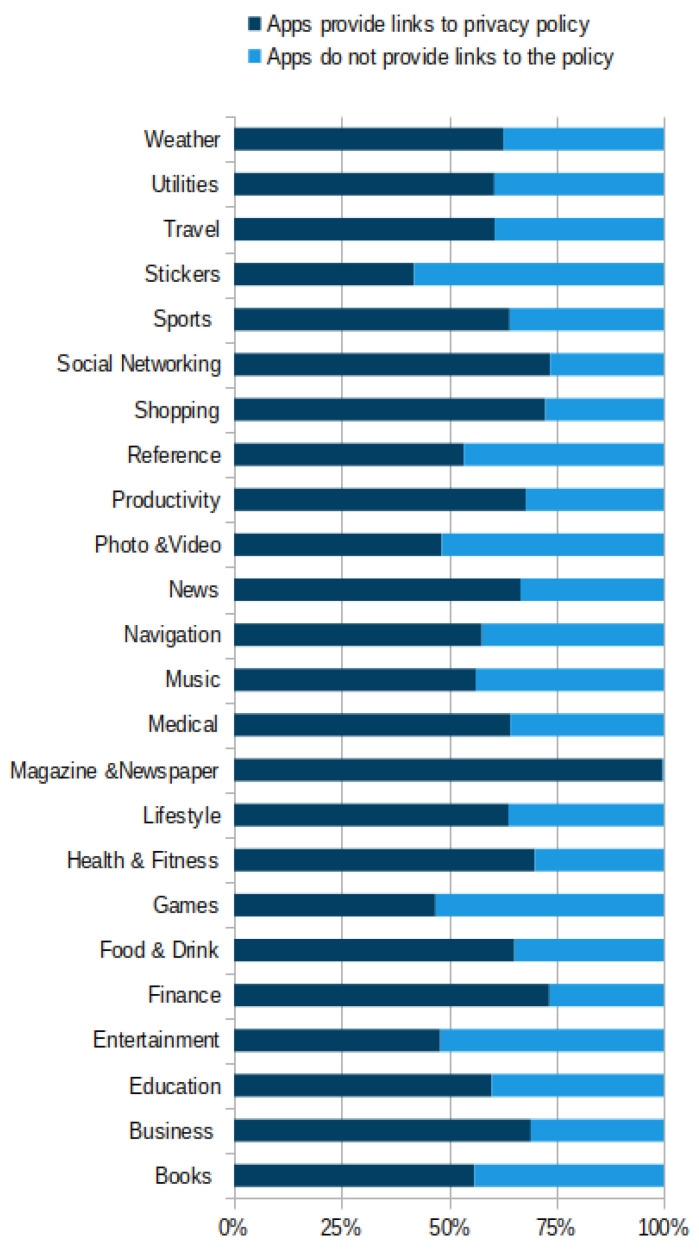
The availability of privacy policy link across the different app categories.

As displayed in Figure 6, our analysis reveals that, among all app categories, Magazine&Newspaper category has the highest percentage of providing policy links with about 98% of its apps offering links to their policies, followed by Finance and Social Networking categories with around 70% of their apps submitting policy links. However, our analysis also shows that the majority of apps in the Stickers category, around 57%, have no policy links, followed by Entertainment, Games, and Photo&Video where around 50 percent of their apps were found to be without any privacy policy links at all. The results also show that on average, the percentage of apps that offer policy links is about 61% across all app categories, while the average percentage of ‘not offering links’ to the policies is about 36%. This finding indicates that in most app categories the majority of apps have allegedly provided privacy policy links. In spite of this, there are some app categories which have more than half of their apps without any links to policies, in particular: Entertainment, Games, Photo&Video, and Stickers. It is striking to find that the categories with the highest number of apps, Entertainment with over 222K apps and Games with more than 242K apps, have more than 50% of their apps without links to a privacy policy.

### 5.2. Policy vs. Non-Policy

**Figure 7:**
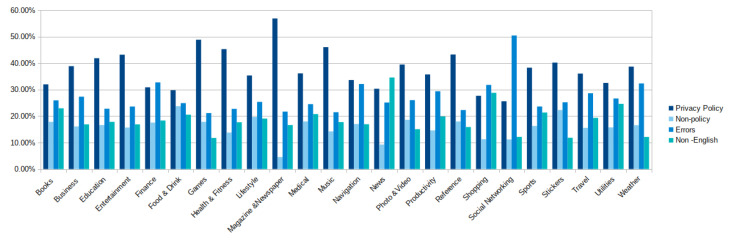
Overview of the broad classes of privacy policy links after investigation.

As discussed in the previous subsection, the majority of apps in the various categories have provided links to policies. Nevertheless, our examination of these links reveals that, when followed, not all of them lead to privacy statements. Figure 7 illustrates the percentage of what we have found after opening each link and inspecting its content. It is surprising, and perhaps worrying, to find that less than half of the apps have privacy policies in almost all app categories. The rest of the categories have approximately between 25 to 45 percent of their alleged policy links direct to privacy policies. On average, only around 38 percent of the links provided by apps lead directly to privacy policies across all app categories. Moreover, the average percentage of broken links (i.e., 404, 403, etc.) across the app categories, seems to be relatively high, as about 26% of available links are not working. The links directing to non-policy (e.g., homepages) and non-English content have an average of about 16 and 18 percent among all the categories.

Our results show that out of 24 different app categories, only one app category, namely, Magazine&Newspaper has above 50% of its apps policy links actually leading to privacy policies and only about 5% leading to non-policy documents. Despite that, the percentages of broken links (22%) and links leading to non-English pages (17%) are still relatively high. One possible reason for this specific category to have a higher percentage of providing policy links (see the above subsection) and availability of actual policies than when compared to the other categories, is that magazines and newspapers are usually run by companies that have the resources (i.e., lawyers and advisers) needed to ensure the availability and compliance of privacy policies with the relevant laws and regulations.

As noted in Figure 7, the Shopping and Social Networking categories are the only categories that have less than 30% of their apps’ policy links actually leading to policies. While the percentage of broken links in the Shopping category constitutes slightly above 30% of the category’s links, the broken links that apps in the Social Networking categories provide, recorded the highest percentage of broken links among all categories. Over 50% of the links were broken. It is striking to discover that out of 34k alleged policy links, approximately 17k links returned errors when opened. These findings are quite concerning since social networking apps are typically designed to share the personal information of their users, such as names, photos, locations, etc. Beside Shopping and Social Networking categories, Finance, Navigation, and Weather categories had more than a third of their supposed policy links return errors when followed. In light of these results, it seems evident that app stores should take into consideration the implementation of automated tools that enable them to validate the correctness of the policy links before they are submitted by app developers. For space limitation, we refer the readers to Figure 7 for more details on the other categories.

## 6. Discussion

Apple has heavily invested to show itself as a privacy-centric company, a stand which gives it an advantage in the market. This research is intended to challenge the previous statement and answer questions related to the status of the privacy policy in the popular Apple iOS App store. The result of our research confirms that Apple is not different from Google when it comes to privacy policy status in their app stores. Neither company enforces systematic reviews of apps’ privacy compliance with the relevant regulatory rules. These rules mandate the requirement of providing real privacy policies describing the actual privacy practices performed by the apps. Instead, both companies allow developers to submit alleged policy links without verifying whether it is a valid link or if it actually leads to the privacy policy. The privacy situation in Apple iOS App Store seems to be a little bit better than Google Play store in terms of providing privacy policy, as the prior work on Google Play store (1 million apps) [38] shows that 50% of apps do not have privacy policies. By contrast, our work (two million apps) shows that only around 38% of app are not providing privacy policy links; however, the significant difference in the numbers of the evaluated apps must be taken into account.

Due to the fact that no previous researchers have conducted similar investigations to ours, in terms of assessing the privacy status in the entire app store, and comparing all the different app categories, the chances of comparison are quite limited. In response to the second question, as we reported in the results section, the majority of the purported privacy policies links did not actually lead to real privacy policies when followed. As a matter of fact, providing a broken link or putting a URL leading to the home page of the app’s owner in the designated place for the privacy policy, instead of putting it in the designated place for the homepage, raises a serious question about the consideration of users’ privacy by the app developers. This situation may indicate that the app developers have not considered writing a privacy policy describing the practices followed by their apps. In addition, without a privacy policy, neither users nor the experts in the field would know what information is collected by the apps and how it is handled and used, not to mention any rights the users may have. Lastly, we found that a considerable number of links have led to non-English web pages. It is worth noting that we do not count the non-English documents for the unavailability of privacy policies as the policies might be written in non-English. However, this language issue is not a major problem as we are only interested in studying the availability of English privacy policy documents, since we are conducting our research in a country that has its vast majority of people speaking native English.

Furthermore, our comparison between the different app categories, in terms of the availability of privacy policy links, shows a clear differentiation between the app categories when it comes to providing links to the privacy policy. For the app categories that have the highest number of apps offering a link to a privacy policy, in particular, Magazine&Newspaper, Finance, and Social Networks, the presence of a link to the company’s privacy policies may play a role in building trust with users and lending credibility to the companies, which is essential for companies in this field. On the other side, users of Games and Entertainment apps, the categories with the greatest number of apps lacking a privacy policy, may be less concerned about privacy or may not have a concept of privacy at all, if we consider children users. Moreover, apps in these categories may not require sensitive information to be provided in contrast to other apps such as Social Networks apps.

Interestingly enough, our research suggests that, in general, if a user downloaded an app from the Apple App Store, it is likely that the apps will provide a privacy policy link. What is important, however, is whether or not this link leads to a privacy statement. Our analysis shows that the overall likelihood that the link provided by any app would take its user to a privacy policy is low; thus, it is unlikely that the link will go direct to a privacy policy page. Although there is a significant chance these links leading to privacy policies may vary from one app category to another, the probability of finding/connecting with privacy policies is low in all app categories.

## 7. Conclusions

In this study, we tackled one of the concerning matters in App stores, the privacy policy. We first introduced the App Privacy Policy Extractor APPE, the system that crawled the entire iOS app store and retrieved the metadata of over two million apps. Our analyses of the metadata showed that more than a third of apps have no privacy policy links and nearly two-third of the apps have provided unverified privacy policy links. After following and examining the content of every provided link to the privacy policy, we found that a substantial number of the links were, in reality, invalid. We also found that a considerable proportion of the links also led to non-policy web pages and non-English pages. Furthermore, from the verified privacy policy links, we constructed the APPC-451K, the most extensive app privacy policy corpus in the literature. It consists of over 451K privacy policy documents. The corpus will be available for the research community to be used for further analysis of apps’ privacy policies using machine learning or other NLP techniques.

## Figures and Tables

**Table 1:** The performance metrics of all DL methods used in PPR development.

	LSTM	LSTM-Att	CNN	RCNN
Accuracy	**95.56**	95.25	95.02	95.11
Recall	**96.69**	96.15	96.37	96.09
Precsion	**95.5**	92.97	94.34	94.4
F1	**95.82**	94.79	95.23	94.21

**Table 2:** The most frequently encountered errors by the APPE.

Error	Occurrence
404 Not Found	124,533
DNS Lookup	113,081
Timeout	35,352
403 Access Denied	20,129
500 Internal Server Error	5634
503 Service Unavailable	3108
410 Gone	1863
502 Bad Gateway	856
400 Bad Request	785
451 Unavailable For Legal Reasons	481

## Data Availability

The data (APPC-451 corpus) are available from the authors upon request. https://github.com/mrhamry/APPE, accessed on 29 September 2022.
